# Supplementary material for: Tissue volume estimation and age prediction using rapid structural brain scans
Source: Sci Rep. 2022 Jul 14;12:12005. doi: 10.1038/s41598-022-14904-5 (PMC9283414; doi:10.1038/s41598-022-14904-5)
Supplement: Supplementary file 1 — Supplementary Information. [file 41598_2022_14904_MOESM1_ESM.pdf]

Supplementary Information for: Tissue volume estimation and age prediction using rapid structural brain scans

Harriet Hobday*, James H. Cole, Ryan A. Stanyard, Richard E. Daws, Vincent Giampietro, Owen O'Daly, Robert Leech, František Váša*

*Correspondence: Harriet Hobday and/or František Váša, Institute of Psychiatry, Psychology & Neuroscience (IoPPN), Academic Neurosciences Centre (PO43), De Crespigny Park, London, SE5 8AF, United Kingdom

Email addresses: harriet.hobday@kcl.ac.uk (Harriet Hobday), fdv247@gmail.com (František Váša)

		EPImix T_1-w VS T_1-w	EPImix T_1-w VS T_1-w FoV_{EPI}	T_1-w VS T_1-w FoV_{EPI}
95% CI	Δ Spearman r_s	[-0.31, -0.024]	[-0.29, -0.055]	[-0.16, 0.14]
	Δ r^2	[-0.031, 0.15]	[0.015, 0.15]	[-0.051, 0.11]

Table S1: Differences between associations of GM volume with age across contrasts. Bootstrap sampling of participants with replacement was used to calculate differences in the strength of association of GM volume with age between pairs of contrasts. Values correspond to 95% confidence intervals of the difference between corresponding statistics, obtained from 10'000 samples with replacement of 64 participants each. Within each column, differences were calculated by subtracting the statistic for the second contrast from the first; for example, for Δ Spearman r_s between EPImix T_1-w and T_1-w contrasts (i.e. first row and first column), $\Delta = r_s$(EPImix T_1-w) - r_s(T_1-w).

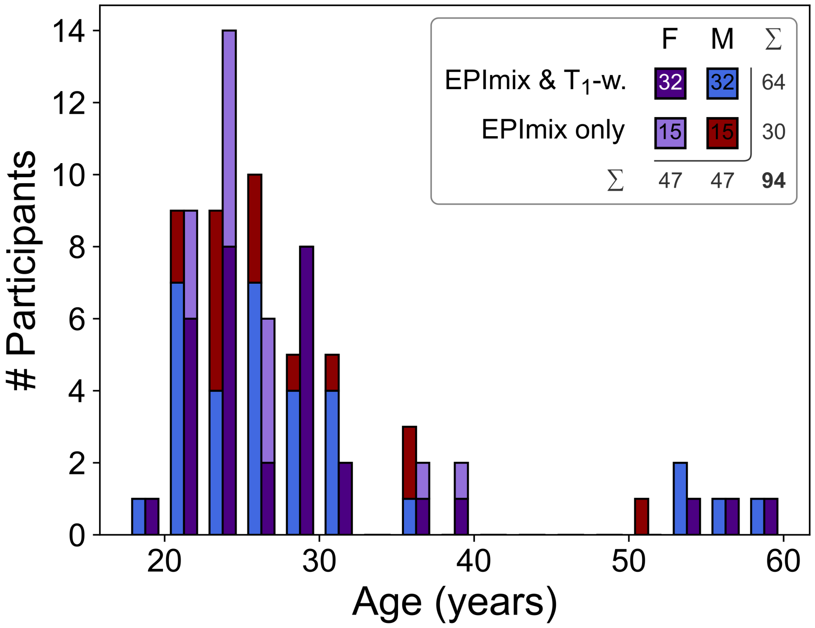

Figure S1: Age distribution of participants by sex and scan sequence. Scans from a total of 94 participants (47 female, 47 male) were included in this study. Of those, 64 (32 female, 32 male) were scanned using both EPImix and single-contrast T_1-w sequences, while an additional 30 (15 female, 15 male) were scanned using EPImix only. There were no differences in participant numbers by sex or scan sequence (Chi-squared test, $\chi^2 = 0$, P = 1).

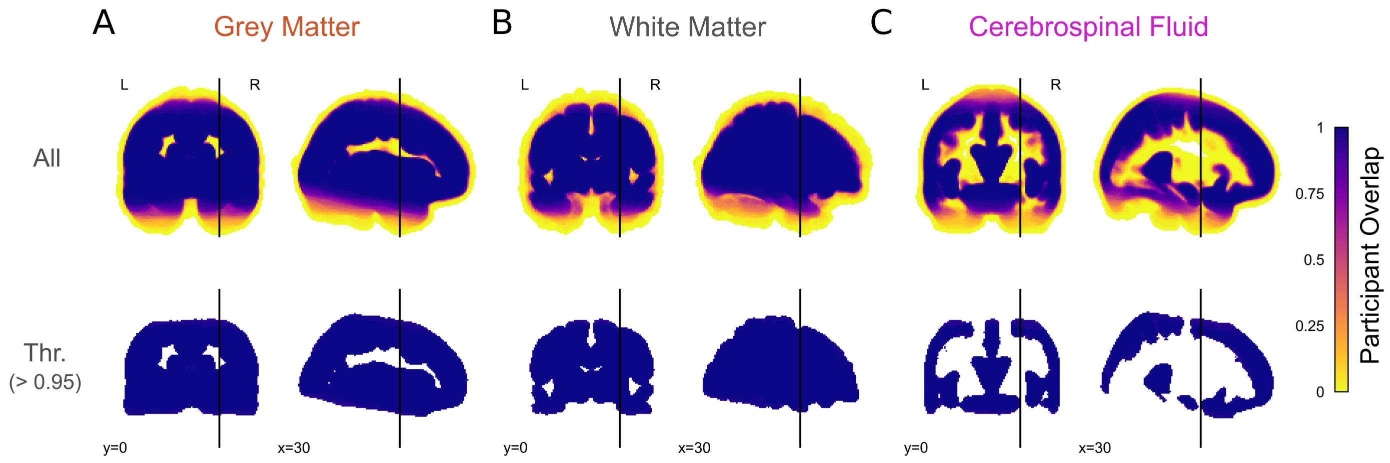

Figure S2: Participant overlap for each tissue type in EPImix scans with reduced FoV. Top row: Proportion of participants with at least 0.001 mm^3 tissue volume at each voxel of A) grey matter, B) white matter and C) cerebrospinal fluid. Subsequent analyses were limited to voxels with at least 95% participant overlap (bottom row).

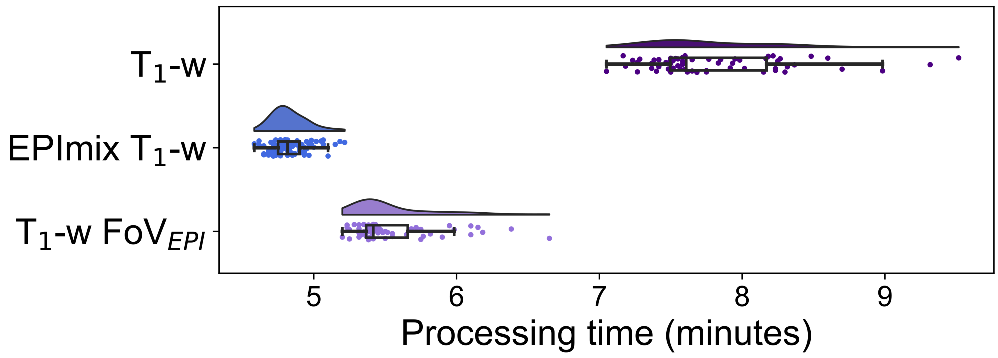

Figure S3: Total processing time (in minutes) for standard T_1-w scans, EPImix T_1-w scans and T_1-w scans with reduced field-of-view.

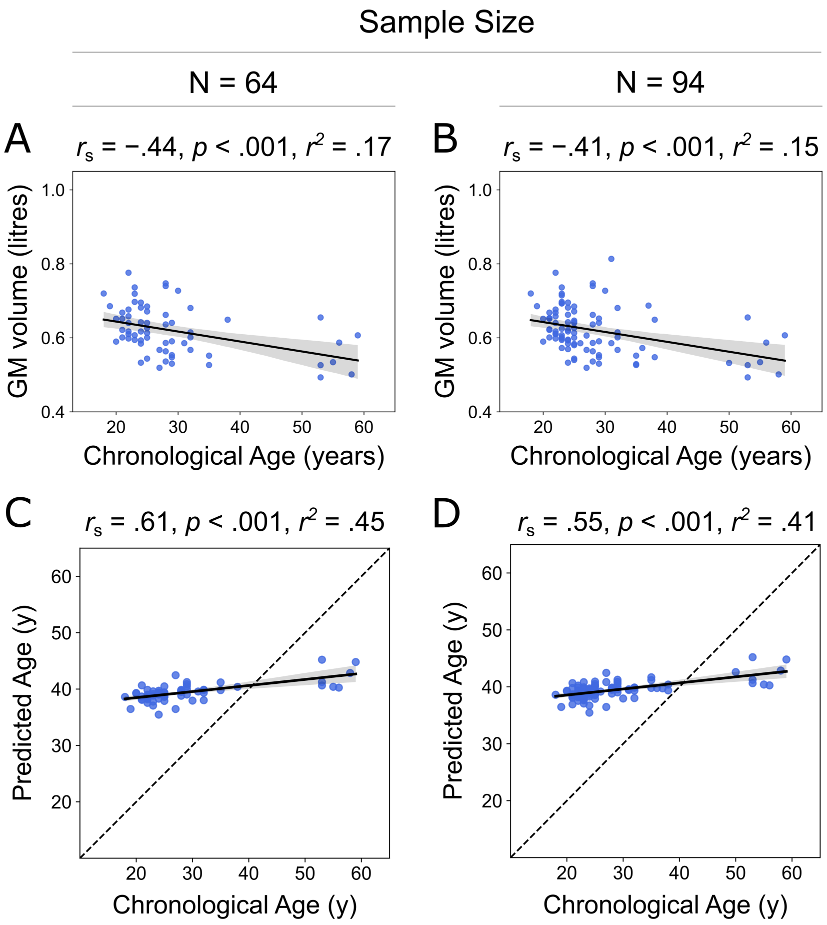

Figure S4: Consistency of EPImix results across sample sizes. Top row: GM volume as a function of chronological age, in A) the subset of 64 participants with both EPImix and single-contrast T_1-w scans, and B) the full sample of 94 participants with EPImix scans. Bottom row: Predicted age as a function of chronological age, in C) the subset of 64 participants with both EPImix and single-contrast T_1-w scans, and D) the full sample of 94 participants with EPImix scans. (Note that panel A is identical to main text Figure 3B, and panel C is identical to main text figure 4B.)
